# Blood Culture–Negative Endocarditis, Morocco

**DOI:** 10.3201/eid2311.161066

**Published:** 2017-11

**Authors:** Najma Boudebouch, M’hammed Sarih, Abdelfattah Chakib, Salma Fadili, Drissi Boumzebra, Zahira Zouizra, Badie Azamane Mahadji, Hamid Amarouch, Didier Raoult, Pierre-Edouard Fournier

**Affiliations:** Institut Pasteur du Maroc, Casablanca, Morocco (N. Boudebouch, M. Sarih);; Centre Hospitalier Universitaire Ibn Rochd, Casablanca (A. Chakib, S. Fadili, B.A. Mahadji);; Centre Hospitalier Universitaire Ibn Toufail Marrakech, Marrakech, Morocco (D. Boumzebra, Z. Zouizra);; Faculté des Sciences Ain Chock, Casablanca (H. Amarouch);; Aix-Marseille Université, Assistance Publique-Hôpitaux de Marseille, Marseille, France (D. Raoult, P.-E. Fournier)

**Keywords:** blood culture–negative endocarditis, Morocco, Casablanca, Marrakech, diagnostic strategy, endocarditis, bacteria, *Bartonella* *quintana*

## Abstract

We investigated the microorganisms causing blood culture–negative endocarditis (BCNE) in Morocco. We tested 19 patients with BCNE by serologic methods, molecular methods, or both and identified *Bartonella quintana*, *Staphylococcus aureus, Streptococcus equi*, and *Streptococcus oralis* in 4 patients. These results highlight the role of these zoonotic agents in BCNE in Morocco.

Blood culture–negative endocarditis (BCNE) can occur when the patient has previously received antibiotic drugs or in the presence of slow-growing or intracellular microorganisms (*1**,**2*). In Morocco, epidemiologic data on endocarditis are fragmentary and show that this disease is frequently associated with rheumatic heart disease (*3*). The aim of this multicenter preliminary study was to investigate the microorganisms causing BCNE in Morocco by using a multimodal strategy.

During June 1, 2009–September 1, 2010, we prospectively included all patients with BCNE seen in 3 health centers in the Morocco regions of Casablanca and Marrakech. The specimens were then referred to the Institut Pasteur du Maroc in Casablanca before being transported to Marseille, France, where diagnostic assays were performed as described by Fournier et al. (*4*). For each studied patient, the physician in charge completed a questionnaire. Answers were not obtained for all questions from all patients.

We used indirect immunofluorescence assays to detect significant levels of antibodies to *Coxiella burnetii* IgG titer to phase I ≥1:800), *Bartonella quintana, B. henselae* (IgG titer ≥1:800), and *Legionella pneumophila* (total antibody titer >1:256), as previously described (*4*). We detected specific antibodies to *Brucella melitensis* by using an immunoenzymatic antibody test (titer >1:200) and to *Mycoplasma pneumoniae* by using the Platelia *M. pneumonia* IgM kit (Bio-Rad, Marnes-la-Coquette, France). When results of first-rank tests were negative, we systematically performed the Western blot test by using *Bartonella* antigens (*4*).

We extracted bacterial DNA from excised valves or EDTA blood by using the QIAmp Tissue kit (QIAGEN, Hilden, Germany) and performed PCR (*5*). We examined paraffin-embedded heart valves and used hematoxylin and eosin stain for histopathologic features (*5*). To detect microorganisms within tissues, we systematically performed the Giemsa, Gram (Brown-Brenn and Brown Hopps), periodic-acid Schiff, Grocott-Gomori, Warthin-Starry, Gimenez, and Ziehl-Nielsen stains (*4*). For patients for whom results of all other techniques remained negative, we performed autoimmunohistochemistry as described by Lepidi et al. (*6*).

Our prospective study enabled the identification of 19 patients (Table): 11 men, 7 women, and 1 person of unspecified gender. Mean age was 40.26 years (range 22–57 years). Among these, 6 lived in urban areas (socioeconomic conditions unknown) and 7 in periurban communities under conditions of poverty. No information on residential environment could be obtained for the remaining 6 patients. All patients except 1 had received antibacterial drugs before blood sampling. 

**Table:** Demographic and clinical features of patients with blood culture–negative endocarditis included in the study.*

Features	Value	% Patients
Sex		
M	11	61%
F	7	38%
Unspecified	1	
Mean age, y	40.26	
Valve involved		
Native valve	18/19	94.7
Valvular bioprosthesis	1/19	5.3
Aortic	4/17	23.5
Mitral	10/17	58.8
Aortic and mitral	1/17	5.9
Tricuspid	1/17	5.9
Echocardiographic signs of endocarditis		
Left-sided endocarditis	2/17	11.8
Right-sided endocarditis	10/17	58.8
Pacemaker infection	1/17	5.9
Valvular vegetation	12/17	70.6
Valvular abscess	6/17	35.9
Clinical symptoms		
Fever (temperature >38.5°C)	17/17	100
Cardiac murmur	16/17	94.1
Glomerulonephritis	1/17	5.9
Other data		
Drug abuse	1/17	5.9
Alcohol dependence	3/17	17.6


*Values are no. patients/no. for which data were available except as indicated.

Samples from all 19 BCNE patients were tested by serologic methods, molecular methods, or both. Among these, we identified an etiologic agent for 4 patients. A 48-year-old man living in impoverished conditions had a positive *Bartonella* serologic test result (IgG 1:6400); Western blot analysis of the serum sample resulted in the specific diagnosis of *B. quintana* infection. In addition, 3 cardiac valves from 3 patients tested by using 16S rRNA PCR were positive, 1 each for *Staphylococcus aureus, Streptococcus equi*, and *Streptococcus oralis*. PCR performed with a second gene confirmed all 3 PCR results. None of these 3 patients lived in impoverished areas.

In Morocco, cases of BCNE represent two thirds of all cases of infectious endocarditis and constitute a major problem of diagnosis and management of patients (*1*). In other countries, the negativity of blood cultures in BCNE may be explained mostly by the administration of antimicrobial drugs before blood culture collection or by the causative role of fastidious microorganisms, as is the case for zoonotic pathogens, such as *C. burnetti*, *B. quintana*, and *B. henselae* (*7*). However, in Morocco, cases of endocarditis caused by these zoonotic pathogens are poorly diagnosed. Previous studies have demonstrated the role of *C. burnetii*, but we could find no study that directly detected *Bartonella* in BCNE. 

In the neighboring country of Algeria, *B. quintana* is considered to be the most common agent of infectious endocarditis, with a prevalence of 15.6%, compared with 5% in countries in Europe (*8*). This difference is likely explained by differences in living conditions (*9*). *B. quintana* infections occur preferentially in disadvantaged populations (homeless) infected by body lice. In our study, 7 patients for whom information on their residential environment was available lived in conditions of poverty and poor hygiene, including the patient who had *B. quintana* endocarditis.

*Bartonella* spp. can infect any heart valve and cause destructive valvular lesions often requiring surgical replacement. Despite the high antimicrobial drug susceptibility of these bacteria, the mortality rate from *Bartonella* endocarditis may reach 31% (*10*). In addition, the recommended treatment for these infections is a combination of doxycycline and gentamicin, which is different from that for endocarditis caused by other common bacterial species, and therefore a specific diagnosis is highly desirable (*10*). However, the small number of patients included in our study precludes the issuance of a definitive recommendation to include serologic testing, molecular testing, or both for zoonotic pathogens in patients with endocarditis in Morocco.
